# Supplementary material for: An open-source framework for large-scale, flexible evaluation of biomedical text mining systems
Source: J Biomed Discov Collab. 2008 Jan 29;3:1. doi: 10.1186/1747-5333-3-1 (PMC2276192; doi:10.1186/1747-5333-3-1)
Supplement: Additional file 1 — Evaluation platform source code. The additional file contains the software evaluation framework discussed in this paper. The most current release can be downloaded from SourceForge.net [1]. See the accompanying README file for instructions on installation and use. Also included in the distribution is a collection of UIMA wrappers for some commonly used BioNLP tools and annotated corpora. [file 1747-5333-3-1-S1.zip › bionlp_uima_repository-1.0.1/docs/api/src-html/edu/uchsc/ccp/util/nlp/tokenizer/Tokenizer.html]

```
001    package edu.uchsc.ccp.util.nlp.tokenizer;
002    
003    import java.util.List;
004    
005    import edu.uchsc.ccp.util.nlp.annotation.TextAnnotation;
006    
007    /**
008     * This class is an interface for tokenizers
009     * 
010     * @author William A Baumgartner, Jr.
011     * 
012     */
013    public interface Tokenizer {
014    
015        /**
016         * Given a <code>String</code> of input text, and a documentID, return tokens in the form of <code>TextAnnotation</code> objects.
017         * 
018         * @param inputText
019         *            the text to tokenize
020         * @param inputDocumentID
021         *            an identifier for the text being tokenized. This document ID will be associated with each of the <code>TextAnnotation</code>
022         *            objects that are returned.
023         * @return a List of <code>TextAnnotation</code> objects, one for each token
024         */
025        public abstract List<TextAnnotation> getTokensFromString(String inputText, String inputDocumentID);
026    
027    }
```
